# So You Think You’re Reflexive? A Modern Approach to Reflexivity in Medical Education

**DOI:** 10.15694/mep.2020.000206.1

**Published:** 2020-09-25

**Authors:** Hoi Yan Corliss Wong, Wendy Lowe

**Affiliations:** 1 Li Ka Shing Faculty of Medicine; 2 Barts and The London School of Medicine and Dentistry

**Keywords:** reflexivity, reflection, social media, social awareness, equity

## Abstract

This article was migrated. The article was marked as recommended.

Reflection is now considered a hallmark of good medical practice. The incorporation of reflective student assignments and activities across medical curricula worldwide are a collective nod to the essentiality of reflection in medicine. However, when the link between reflective and clinical practice fails to be established or emphasised, students are sometimes left wondering what purpose their reflection serves. Consequently, superficial engagement, or even outright disengagement can ensue.

In this paper, a case is made for reflexivity, reflection’s socially oriented cousin, to be introduced as part of formal medical training. The proposed approach utilises the pedagogical value of social media and builds upon the burgeoning social awareness of the latest generation of medical students to incite actionable change. It is suspected that by offering a more timely, challenging, and engaging learning experience, students will begin to understand the necessity of personal introspection, and the duty they have in the delivery of equitable healthcare.

## Introduction

Reflection is defined as a goal-oriented process in which the aim is to critically inquire, evaluate and re-examine practice ( Barrett, Kajamaa and Johnston, 2020). Lately, it has grown to assume an established role in medical education, propelled by a rising recognition of the need to cultivate self-aware healthcare practitioners. Its purported benefits are wide-ranging, spanning from bridging the theory-practice gap to encouraging critical problem resolution ( Paterson and Chapman, 2013). However, as an intercalating medical education student armed with pedagogical knowledge in one hand and the medical student experience in the other, I have come to question the effectiveness of current teaching on reflection in meeting the demands of tomorrow’s patients. In this article, I draw inspiration from two drastically different sources: my experience as an external student from Hong Kong, and social media to propose a novel approach to developing reflexive, as opposed to reflective, practice.

## Personal Reflexive Experience

As part of my current course, I have been teaching clinical skills to first year medical students. It has certainly been very eye-opening to witness not only how medicine is taught on opposite sides of the globe, but how it is received and taken forward by its students. Personally, I feel there is no better setting than the clinical skills environment to observe these differences, especially where culture is concerned. For instance, at my home institution, only males are invited to be examined on for clinical skills demonstrations requiring chest exposure. In contrast, I was explicitly asked by my British supervisors to extend the invitation to all of my students, which was initially quite shocking to me.

When asking for volunteers, there was substantial reflection-in-action going on as my conservative Eastern upbringing locked horns with the more liberal attitudes of the West. I was driven to encourage my female students to volunteer, but simultaneously held back by deeply ingrained societal norms and attitudes that police the female body and dictate levels of appropriate exposure. Without a doubt, there were times I felt guilty in feeling a sense of relief I was not in their shoes.

By eventually extending my reflection to involve challenging certain cultural norms and my own discomfort relating to appropriate female body exposure, I found myself far more ready to volunteer if the occasion were to arise. I began contemplating writing to the clinical skills team back at home to have this issue addressed. Needless to say, it took me multiple cycles of reflection to acknowledge how my upbringing has influenced my attitudes and behaviours.

I slowly began to recognise medical school culture as a culture in its own right, one that starts off as a microcosm of wider society before diverging over time. I found solace in medicine’s anatomical view of the human body, as opposed to an aesthetic one carved by societal pressures and social media. It also helped to think of the established standards of professionalism that render the clinical skills setting far safer and less judgemental than most. On top of this, by being on the other side of the teacher-student relationship, I came to understand the educational benefits of being peer examined. Nevertheless, I firmly believe reflexivity had a major role to play in overcoming the unease I felt. Now that I am more attuned to how my culture has shaped my identity, I consider myself better placed to educate my future students and treat my future patients.

I first came across the concept of reflexivity in a practitioner research seminar. While reflection and reflexivity are sometimes used interchangeably, the latter involves interpretation on top of repeated cycles of reflection. What this means is that reflexive practice extends beyond thinking of certain experiences, instead encouraging the practitioner to contemplate how their outcomes of personal introspection may relate to their own sociocultural locations ( Landy *et al*., 2016). One may even choose to take reflexivity one step further and relate it to equity and justice. Here, the idea is to recognise privilege and oppression such that the practitioner is better equipped to take action and challenge the status quo ( Landy *et al*., 2016).

It concerns me that medical students may go through training without ever seeing the need to question their own assumptions and values. When hard facts and strict protocols disproportionately comprise the medical education experience, they may be deceived into thinking they are acting fairly when they are not. For example, a study revealed medical students were more likely to negatively stereotype obese patients, anticipate lower adherence and show less visual contact compared to non-obese patients ( Persky and Eccleston, 2010). Based on these findings, it was recommended that the nature and origin of biases be identified to improve rapport within the clinical encounter. Identification is merely the first step of many towards the provision of truly equitable healthcare, but even this step can prove challenging to students if they do not know where or how to look.

Medical educators must not forget how difficult a process personal introspection can be, not just in its initiation but in its follow-through with no end-goal in sight. Reflection, and indeed reflexivity, do not necessarily come naturally to most medical students. To prevent superficial commentaries on the mundane, medical educators can adopt a facilitative role to kickstart the reflexive process, but how so?

## Modern Solutions

A quick Google search tells me I belong to Generation Z, born and raised with social media. While I admit I have spent countless nights app-switching and scouring the Internet, I would like to think these hours have not gone to waste. Courtesy of YouTube’s algorithms, I was recommended a video called ‘Do You Have a Racial Bias’ several years ago. In the video, participants of different races were invited to take the Black-White Implicit Association Test and discuss their results. More recently, I have tuned in to a series called ‘Spectrum’ by Jubilee. In each episode, participants who share commonalities, such as race, illnesses, and occupations, are asked questions sent in by viewers to see whether they think the same way. After each question is asked, participants either physically or virtually move along a spectrum from strongly agree to strongly disagree and are invited to justify their opinions in a group discussion. Since their release, millions upon millions of viewers have watched these videos.

Beyond their evident entertainment value, I feel both of these ideas have high pedagogical potential. The familiarity of social media may ease Generation Z medical students like myself and the even more Internet-agile generations to follow into the foreign territory of reflexivity. With a little tweaking, I can envision these activities integrated into a variety of settings, including Zoom seminars and tutorials, or in-person clinical skills sessions once face-to-face teaching returns. Regardless of the context in which it is implemented, the underlying rationale is the same. The idea is to create safe, yet engaging spaces in which educators facilitate the transfer of the previously implicit into students’ consciousness through disorienting dilemmas. For some students, it may be the first time they receive a definite confirmation of their subconscious individual differences, or the first time they have been able to discuss their own experiences in the medical context. The ultimate goal is for students to eventually be able to carry out the entire process themselves: starting by identifying individual differences and overcoming possible cognitive dissonance, to exploring their origins and finally, use what they have uncovered to shape their practice.

At the moment, reflexivity is yet to enter the mainstream lexicon of medical education. However, its introspective depth, recursive nature and sociocultural directionality arguably make it a significant improvement from reflection. Like other skills, reflexivity needs to be learnt, practised, and applied. Inspiration may be drawn from social media, and also furthered by it, to get the message across.

Through social media, I have witnessed fellow medical students rallying behind the social justice movement Black Lives Matter. A petition to include diversified case representations in clinical teaching, particularly in instances where diseases may present differently between races, has garnered over 200,000 signatures ( K, 2020). It is evident there is a need for change. More importantly, efforts like this indicate there are already medical students who possess the resourcefulness, fervour, and tenacity of agents of change.

One of the greatest challenges in healthcare today is attending to diversifying patient populations with increasingly varied needs. To overcome deep-seated social injustices, we require doctors who understand patients, except the pre-requisite is that doctors must understand themselves first. It took being displaced 6000 miles from my conservative culture for me to do so. With social media, medical educators can now recreate this learning experience in situ, and help students channel their social justice energy into shaping the medical landscape of tomorrow. After all, modern problems require modern solutions. A contemporary take on reflexivity may very well be the solution medicine needs.

## Take Home Messages

• Reflection alone may be inadequate to meet the demands of future patient populations.
• Reflexivity is a skill that should be taught as part of formal medical training.
• Medical educators should seek to create enriching and cognitively dissonant learning environment.
• There is substantial, untapped pedagogical potential in modern media.
• Concrete steps should be taken towards the delivery of socially just healthcare.

## Notes On Contributors

Hoi Yan Corliss Wong has recently completed her BSc in Medical Education at Barts and will be resuming her medical studies at Li Ka Shing Faculty of Medicine. She currently works at MedEd Lab HK, a virtual student-centred laboratory that focuses on artificial intelligence systems and peer-assisted learning research. Her ORCID number is https://orcid.org/0000-0001-7619-7821.

Wendy Lowe is a Senior Lecturer in Sociology and Medical Education at Barts. Her educational research interests include exploring sociocultural learning environments, in addition to broadening participation and social capital within medical institutions. Her ORCID number is https://orcid.org/0000-0003-3804-4263
