# Supplementary material for: So You Think You’re Reflexive? A Modern Approach to Reflexivity in Medical Education
Source: MedEdPublish (2016). 2020 Sep 25;9:206. [Version 1] doi: 10.15694/mep.2020.000206.1 (PMC10699397; doi:10.15694/mep.2020.000206.1)
Supplement: Peer review history

# Reviewer report: Reviewer response for version 1

**DOI:** 10.21956/mep.20189.r31230

**Published:** 2020-12-12

**Authors:** Megan Anakin

**Affiliations:** 1 University of Otago

This review has been migrated. The reviewer awarded 4 stars out of 5 The word ‘reflexivity’ caught me in the title and by the time I read the abstract, I knew I was going to be reading very thought-provoking article. The introduction prepares the reader to engage with the well-described personal reflexive experience and to contemplate a number of modern solutions to introduce the concept to medical students. I appreciate the first author’s voice and perspective is emphasised in this article. To develop these ideas further, please consider exploring the medical education (and higher education) literature further with support from the senior author as a guide. By making greater use of the literature, you may be able to further develop your argument and find evidence to support the solutions you suggest. I look forward to reading the revised version of this article to see how you have developed your ideas. Have you considered designing a study to investigate medial students’ (and their educators’) conceptions of reflexivity? I think you have opened up a conversation about a fascinating topic in this journal. Bravo!

Reviewer Expertise:

NA

No decision status is available

# Reviewer report: Reviewer response for version 1

**DOI:** 10.21956/mep.20189.r31229

**Published:** 2020-10-03

**Authors:** Enoch Chan

**Affiliations:** 1 The University of Hong Kong

This review has been migrated. The reviewer awarded 4 stars out of 5 This is a great article highlighting the importance of reflexivity in one’s development as a medical practitioner and medical educator. The account of the author’s own reflexive experience certainly provided a strong testimony of how a clash of worldview in clinical encounters helps a medical student challenge their own beliefs and bias, and from there develop as a medical practitioner.Here are some considerations/suggestions for the author to make a stronger case for reflexivity to be added to a medical curriculum:The authors could perhaps discuss about their person experience with how reflexivity and cultural bias are being addressed in their institutes. This will help readers gain more insight about whether this is lacking (or adequate?).The author has provided an example of how cultural bias is evident amongst medical students. Perhaps some additional examples on how this is relevant relating to patient care (e.g. breaking bad news to terminally ill patients, dealing with family members of patients who requested DNR or DNACPR etc.).

Reviewer Expertise:

NA

No decision status is available

# Reviewer report: Reviewer response for version 1

**DOI:** 10.21956/mep.20189.r31231

**Published:** 2020-09-28

**Authors:** Siobhan McHugh

This review has been migrated. The reviewer awarded 3 stars out of 5 This is a piece of writing that raises a number of key issues in the progression of medical education, and I certainly enjoyed the personal reflections throughout. One of the real strengths of the piece is the clear distinction between reflection and reflexivity. This is apparent not only in the recognition of reflexivity placing the practitioner more wholly within the sociocultural domain, but also in the assertion that the real strength of reflexivity is in making what is inherent, apparent. The topic is very well linked to medical education, and makes a clear argument for innovative pedagogy. In fact generally the article includes a relatively good balance of personal reflection and relevant academic information. However, I would have liked to see more specific reference to the use of reflexivity in applied health research. Although the author is correct that reflexivity is still under-utilised, if utilised at all, in medical education, there is a wealth of literature in which reflexivity has been used in various ways to prompt learning and improvement in clinical practice. I would perhaps have liked to see a clearer presentation of how social media could be used in a practical way to prompt reflexivity and reflexive discussion between medical educators and their students, and this literature may have provided some ideas.The target audience for this piece are arguably medical educators and those with the capacity to develop medical education, however the thoughtful personal contributions in this piece would prompt learning for all who read it. There is real strength in the questions that this piece prompts, and it would be very interesting to see a more detailed piece of work outlining some clear and practical ideas to embed reflexivity in medical education on the back of a very thoughtful and personal piece of work.

Reviewer Expertise:

NA

No decision status is available
